# Optimal fine ϕ-slicing for single-photon-counting pixel detectors

**DOI:** 10.1107/S0907444911049833

**Published:** 2011-12-09

**Authors:** Marcus Mueller, Meitian Wang, Clemens Schulze-Briese

**Affiliations:** a Swiss Light Source at Paul Scherrer Institut, CH-5232 Villigen, Switzerland

**Keywords:** diffraction data collection, data-collection strategies, detectors, hybrid pixel detector, single-photon counting

## Abstract

The data-collection parameters used in a macromolecular diffraction experiment have a strong impact on data quality. A careful choice of parameters leads to better data and can make the difference between success and failure in phasing attempts, and will also result in a more accurate atomic model. The selection of parameters has to account for the application of the data in various phasing methods or high-resolution refinement. Furthermore, experimental factors such as crystal characteristics, available experiment time and the properties of the X-ray source and detector have to be considered. For many years, CCD detectors have been the prevalent type of detectors used in macromolecular crystallography. Recently, hybrid pixel X-ray detectors that operate in single-photon-counting mode have become available. These detectors have fundamentally different characteristics compared with CCD detectors and different data-collection strategies should be applied. Fine ϕ-slicing is a strategy that is particularly well suited to hybrid pixel detectors because of the fast readout time and the absence of readout noise. A large number of data sets were systematically collected from crystals of four different proteins in order to investigate the benefit of fine ϕ-­slicing on data quality with a noise-free detector. The results show that fine ϕ-slicing can substantially improve scaling statistics and anomalous signal provided that the rotation angle is comparable to half the crystal mosaicity.

## Abstract 2

Fine ϕ-slicing substantially improves scaling statistics and anomalous signal for diffraction data collection with hybrid pixel detectors.

## 1. Introduction

Collection of X-ray diffraction data is the central experiment in the process of crystal structure determination and analysis. The importance of making careful choices for the data-acquisition parameters in order to achieve the best possible data has been discussed in a number of publications (see, for example, Pflugrath, 1999 ▶; Bourenkov & Popov, 2006 ▶; Dauter & Wilson, 2006 ▶; Dauter, 2010 ▶). A good and carefully chosen data-collection strategy that leads to better data can make the difference between success and failure in phasing attempts, and better data will also result in a more accurate atomic model (Jiang & Sweet, 2004 ▶). The selection of data-acquisition parameters has to account for the goal of the experiment, which is usually to apply the data in molecular replacement, anomalous phasing, high-resolution refinement or a ligand search. These applications have different requirements for data completeness, accuracy and resolution (Dauter, 2010 ▶). Furthermore, experimental factors such as the crystal characteristics, the available experiment time and the properties of the X-­ray source and detector have to be taken into account.

For many years, CCD detectors have been the prevalent type of detector used in macromolecular crystallography. Most recommendations for data-collection strategies as well as the experience of experimenters are based on the characteristics of this detector type, as well as the previously used imaging plates. Recently, hybrid pixel X-ray detectors, which operate in single-photon-counting mode, have become available for macromolecular crystallography (Broennimann *et al.*, 2006 ▶; Hülsen *et al.*, 2006 ▶). The commercially available PILATUS hybrid pixel detector is now in standard user operation on an increasing number of macromolecular crystallography synchrotron beamlines. These detectors have fundamentally different characteristics and offer various advantages over CCD detectors (Hülsen *et al.*, 2006 ▶; Tate *et al.*, 2006 ▶). The most important features of hybrid pixel single-photon-counting detectors are as follows. (i) No readout noise and dark current as sources of detector noise. (ii) A sharp point-spread function of one pixel, which results in excellent resolution of closely spaced reflections over the entire dynamic range of the detector and maximizes the signal-to-noise ratio. (iii) A short readout time in the millisecond range. This allows the collection of diffraction data with continuous rotation and eliminates the shutter as a source of error. (iv) A high dynamic range of 20 bits helps to overcome issues with incomplete low-resolution data arising from overloads (Hülsen *et al.*, 2006 ▶).

Photon-counting area detectors have previously been successfully employed in macromolecular crystallography in the form of multiwire proportional counters (Hamlin *et al.*, 1981 ▶; Blum *et al.*, 1987 ▶). The direct quantization in these gas-discharge counters results in very low noise, but their design suffers from poor spatial resolution and, more importantly, low global count rates (up to 20–50 kHz over the whole detector surface) that prohibit their effective use on third-generation synchrotron beamlines. Hybrid pixel detectors offer better spatial resolution and far superior local count rates of up to several Mcps per pixel, without a practical limitation of the overall global count rate.

To fully exploit the advantages of hybrid pixel detectors, different data-collection strategies to those established for CCD detectors need to be applied because of the different characteristics of the two types of detectors. Fine ϕ-slicing has previously been identified as a desirable strategy for data collection, but was not practical for detectors with a long readout time (Pflugrath, 1999 ▶). Hybrid pixel detectors are particularly well suited to fine ϕ-slicing because of their fast readout time and the absence of readout noise. We systematically collected a large number of data sets from crystals of four different proteins in order to investigate the benefit of fine ϕ-­slicing on data quality with a noise-free detector in practice. This study focuses on fine-slicing and the optimization of the rotation width per image for the best overall and highest shell statistics. Other data-collection parameters such as flux and exposure time, which can be optimized to take detector characteristics such as count-rate limitation or read­out time into account, are the subject of separate investigations owing to the complexity of each of these aspects.

## 2. Aspects of fine ϕ-slicing

### 2.1. Accuracy of intensity estimates

The aim of a diffraction experiment is to accurately determine the intensities of Bragg reflections. A peak intensity *P* is composed of the reflection intensity *I* and its background *B*. The observed intensity *I* of a Bragg reflection is the sum of the counts of the pixels in the peak region after subtracting the estimated background of the corresponding peak pixel,
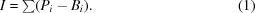
The diffraction of X-rays behaves as a Poisson process. The quantity *Q* of photons counted in a diffraction experiment is Poisson-distributed with variance *Q* and standard deviation σ,
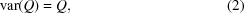

               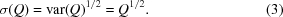
The ratio of intensity to its standard deviation is a criterion for the quality of the measurement,
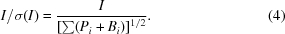
From this equation, it follows that the accuracy of the observed intensity is lower for larger backgrounds and smaller intensities are measured less accurately. Conversely, when a reflection is measured in conjunction with less background its intensity is determined more accurately, and this effect is more pronounced for small intensities.

### 2.2. Qualitative description of fine-slicing

**Figure 1:**
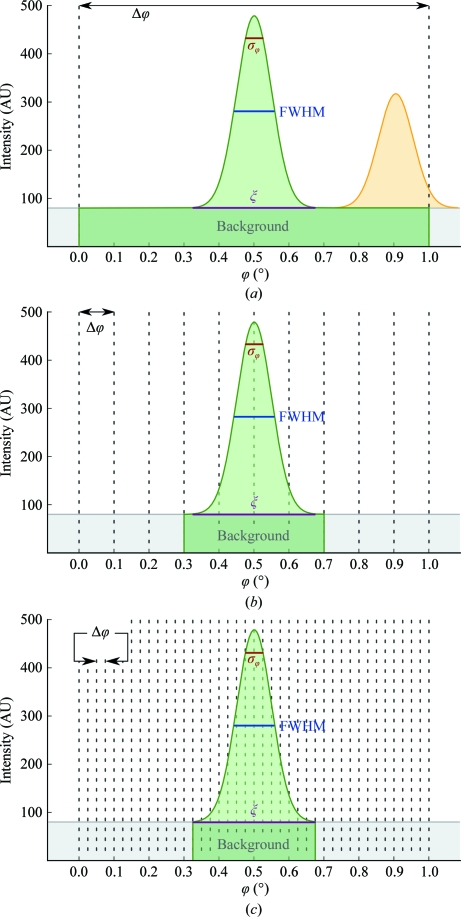
Wide-sliced and fine-sliced data collection. The background and the reflection intensity along ϕ, assuming a Gaussian distribution of the reflection intensity with σ_ϕ_ = 0.05°, are shown. The reflecting range ξ, FWHM and σ_ϕ_ of the reflection are indicated. (*a*) Wide-sliced data collection with a rotation width of 1°. The intensity of a full reflection (green outline) is recorded on a single image without sampling of the profile along ϕ. A large amount of background overlaps with the reflection intensity along ϕ and is included in the integration. A partial reflection (orange outline) is recorded on two consecutive images with twice the background of a full reflection. (*b*) Intermediate fine-slicing at Δϕ = 2σ_ϕ_ = 0.1°: improved background separation and coarse sampling of the profile along ϕ. (*c*) Fine-slicing at Δϕ = 0.5σ_ϕ_ = 0.025°. The reflection profile is densely sampled along ϕ. The inclusion of ϕ regions which contain background but no parts of the reflection profile and intensity in the integration is further reduced.

The angular width of rotation range per image is an important variable when acquiring diffraction data using the rotation method. Based on the relation between reflecting range, which is the angular spread of reflections, and the rotation range per image Δϕ, two basic strategies can be distinguished (Fig. 1 ▶). In a wide-slicing strategy, Δϕ is larger than the reflecting range and most reflections are recorded fully on a single image. The wide rotation range leads to a smaller number of images for a complete data set that need to be read out from the detector, stored and processed. This strategy was frequently chosen in the past because of practical considerations to minimize the acquisition time with slow detectors and when limited storage and computing resources were available. The maximum rotation range per image is limited by the occurrence of overlapping reflections caused by intersecting lunes and can be estimated by the following formula (Dauter, 1999 ▶),
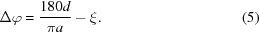
The maximum rotation range per image depends on the high-resolution limit *d*, the reflecting range ξ, and *a*, the length of the primitive unit-cell dimension projected onto the incident X-­ray beam. A small angle between the beam direction and a long unit-cell axis prohibits large rotation ranges. The problem of overlaps from intersecting lunes is less severe in the case of small non-orthogonal unit cells, where rows of reflections from one lune often fit between rows from adjacent lunes without overlap. Nevertheless, overlaps can easily render a wide-sliced data set unusable or strongly degrade its maximum resolution if the rotation range is not carefully chosen.

In a fine-slicing strategy, Δϕ is only a fraction of the reflecting range and the reflection intensities are distributed over several consecutive images. This strategy offers a number of advantages over the wide-slicing approach. Obviously, fine-slicing reduces the problem of overlapping reflections from intersecting lunes. More importantly, in the wide-slicing strategy the reflections are recorded together with background over a wide angular range, whereas the overlap with background along ϕ is minimized with fine-sliced images (see also Fig. 1 ▶). Therefore, in the absence of systematic errors reflection intensities can be determined more accurately, as outlined above.

### 2.3. Profile fitting

Another advantage of fine-slicing is that it leads to better profile fitting, which is the standard technique for integrating reflection intensities from macromolecular diffraction data (Diamond, 1969 ▶; Ford, 1974 ▶; Rossmann, 1979 ▶). A reflection profile describes the shape and the distribution of intensity. Reference profiles can be generated by superposition of nearby reflection peaks. The reference profiles are used to estimate the reflection intensities by a least-squares fit of the observed to the reference profiles. The intensity estimate is derived from a scale factor used in the fitting procedure.

Two- and three-dimensional profile fitting are distinguished based on calculating reflection profiles and intensities per image or over a number of adjacent images. In the case of two-dimensional profile fitting, usually only peaks from the same image are used to calculate reference profiles. Fractional intensity estimates of a partially recorded reflection are calculated for each of its images, and intensities of partial reflections are obtained by summing the estimates after integration. In three-dimensional profile fitting, partial observations of consecutive images are used to reconstruct full reflections. The full three-dimensional profile of a reflection is then fitted against the reference profiles (Kabsch, 1988 ▶). In principle, three-dimensional profile fitting should lead to better intensity estimates than two-dimensional integration (Leslie, 2006*b* ▶). However, to date substantial benefits of three-dimensional profile fitting have not been demonstrated in practice.

Compared with intensity estimation by summation integration, profile fitting reduces the random error in the data set, which is especially advantageous for the determination of weak intensities (Diamond, 1969 ▶; Ford, 1974 ▶; Rossmann, 1979 ▶). The standard deviation of the integrated intensities of weak reflections can be reduced by a factor of 2^1/2^ by profile fitting compared with summation integration (Leslie, 2006*a* ▶). Both the calculation of reference profiles and profile fitting require the superposition of reflections, for which their spot centroids need to be correctly determined. At finer rotation angles, when more images contribute to spot intensities and the spot is better sampled along ϕ, spot centroids can be determined more accurately (Kabsch, 2010*a* ▶). This should improve the accuracy of the intensities estimated by three-dimensional profile fitting.

### 2.4. Quantification of fine-slicing

A qualitative description of fine-slicing is how finely reflections are sampled on consecutive diffraction images. To quantify fine-slicing, it can be expressed as rotation angle in units of reflecting range or mosaicity. Since different definitions of mosaicity are in use, it is important to also consider the mosaicity definition used for quantification. In this paper, we investigate fine-slicing as a function of Δϕ/σ_ϕ_, where σ_ϕ_ is the standard deviation of the reflection profiles assuming a Gaussian distribution, the definition of mosaicity used in *XDS* (Kabsch, 2010*b* ▶). An alternative to the Gaussian model used by *XDS* is a spherical model for reciprocal-lattice points in which the radius of the reflection sphere is a function of mosaicity (Greenhough & Helliwell, 1982 ▶). This spherical model is used, for instance, by *MOSFLM* (Leslie, 2006*b* ▶). Another mosaicity definition is the full-width at half-maximum (FWHM) of the reflection profile, which is approximately 2.35σ_ϕ_ for a Gaussian profile. It should also be noted that throughout this paper mosaicity refers to the width of the reflection profile along ϕ, not to the true mosaic properties of the crystals (Nave, 1998 ▶).

### 2.5. Fine-slicing and detector characteristics

Despite the advantages of fine-slicing, data quality can also be negatively affected by this strategy when the acquisition of each image is associated with any source of error or noise. Detector readout noise is one obvious source of error that degrades data quality when using a fine-slicing strategy: the more images in a data set, the larger the contribution of the detector readout noise. Other per-image-based errors may arise from the crystal goniometer and the X-ray shutter. The crystal rotation has to be perfectly synchronized with the opening and closing of the X-ray shutter. The mechanical shutter and spindle, however, may not work perfectly reproducibly and may introduce so-called ‘shutter jitter’, which is a variation in the exposure time and angular range of each image. Shutter jitter can cause a strong deterioration in the quality of diffraction data (Diederichs, 2010 ▶). Data acquired using CCD detectors are affected by both detector noise and potential shutter jitter, which restricts the applicability of fine-slicing (Pflugrath, 1999 ▶). CCD detectors are also prone to saturated pixels in strong reflections. This problem of overloads can be alleviated by smaller rotation angles because a reflection intensity is split up over several images.

The use of a hybrid pixel detector eliminates detector noise and shutter jitter as the major sources of per-image-based noise. The single-photon-counting detector can be read out without any associated noise. The problem of shutter jitter is eliminated because the fast readout time allows the collection of data during continuous rotation and exposure of the crystal. Instead of opening and closing the shutter for each image, the frames are simply read out from the detector at an interval corresponding to the image exposure time (Hülsen *et al.*, 2006 ▶). However, during the readout of the detector no data are acquired for a few milliseconds and photons from reflections which are in diffracting conditions are not counted by the detector. The detector readout scales the intensities by the relative difference between readout time and exposure time, while the distribution of intensity and the shape of reflection profiles are not affected (Hülsen *et al.*, 2006 ▶). Therefore, the intensity estimates can be correctly determined despite the detector readout and the associated dead time.

Hybrid pixel detectors have a wide dynamic range and saturated pixels are usually not encountered in practice when collecting macromolecular diffraction data. However, the accuracy with which the strongest reflections with several hundreds of thousands of counts in a pixel are measured might be affected by the count-rate limitation inherent to all counting detectors (Gruner *et al.*, 2006 ▶). Fine ϕ-slicing can improve the accuracy of measuring strong reflections because finer sampling leads to a more constant count rate over the rotation angle and improved count-rate correction (Kraft *et al.*, 2009 ▶).

With fast and noise-free pixel detectors it should, in principle, be possible to fully exploit the advantages of fine-slicing. In practice, however, the extent to which the fine-slicing approach can be pursued might be limited by other factors such as the precision of the diffractometer hardware or the handling of ultra-fine-sliced reflection intensities with only a few counts per pixel by the integration software.

## 3. Materials and methods

### 3.1. Crystallization and data collection

Crystals of insulin, lysozyme and thaumatin were grown and cryoprotected as summarized in Table 1 ▶ and described pre­viously (Nanao *et al.*, 2005 ▶). The hexagonal crystal form of thermolysin was obtained and cryoprotected as described by Juers & Matthews (2004 ▶). All crystals were flash-cooled and stored in liquid nitrogen prior to data collection.

All data were collected on beamline X06SA of the Swiss Light Source at the Paul Scherrer Institut (http://www.psi.ch/sls/pxi/). Crystals were kept near 100 K using a nitrogen-gas stream during data collection. Data from insulin, lysozyme and thaumatin were collected at 1.000 Å wavelength and data from thermolysin were collected at the wavelength of the zinc absorption edge at 1.282 Å. The PILATUS 6M (DECTRIS Ltd) detector was operated at a threshold energy of half the X-ray energy for all data sets. Thermolysin data were collected with a detector readout time of 3.5 ms at high gain; all other data were collected with a 5 ms readout time at low gain.

To investigate the influence of rotation width per image on data quality, we collected series of five to seven data sets for which the rotation width increased by a factor of two between each data set in the series. The exposure time was also increased by a factor of two between each data set, while the flux was kept constant. This resulted in a constant rotation speed and dose rate for all data sets in a series, but the relative dead time per image owing to detector readout was larger for the data sets collected at smaller rotation width. The rotation speed can strongly influence the quality of the data (Diederichs, 2010 ▶). Varying the exposure time per image while keeping the rotation speed constant eliminates this effect at the expense of a potential influence of exposure time and relative dead time on data quality.

**Figure 2:**
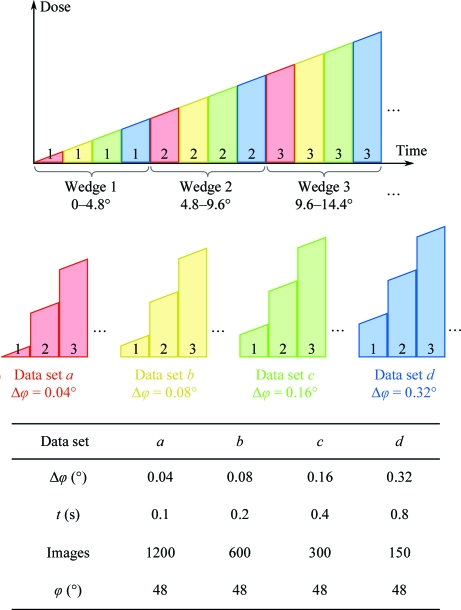
Interleaved data-acquisition scheme. In this example, a series of four data sets *a*–*d* with rotation ranges between 0.04° and 0.32°, exposure times from 0.1 to 0.8 s and a total rotation of 48° covered by each data set is collected at constant flux and dose rate. A first wedge covering 0–4.8° for each of the four data sets is collected, then a second wedge from 4.8° to 9.6° and so on until all ten wedges for the four data sets have been collected. The data sets are then assembled from their wedges, which are discontinuous in time and dose but continuous in rotation. All data sets of the series received a similar dose.

In order to achieve the best possible comparability between the data sets in each series, we took the following measures. (i) All data sets in a series were collected at the same position of a single crystal using the same starting angle. This excludes any effects of heterogeneities in diffraction properties within the same crystal or between different crystals on data quality. (ii) Each series was collected with the same angular speed and dose rate, increasing the exposure time per image according to the increase in rotation width. (iii) Radiation damage was minimized by strongly attenuating the beam and defocusing to a size of 100 × 100 µm. Furthermore, radiation damage was equally distributed over all data sets of a series using the interleaved data-acquisition scheme depicted in Fig. 2 ▶. The total dose for a series of data sets was in the range ∼0.3–8 MGy as estimated with *RADDOSE* (Paithankar & Garman, 2010 ▶). Overall, 16 series consisting of a total number of 94 data sets were used in this study (Table 4). The data sets are available for download at http://www.wuala.com/mueller_et_al/fine_phi_data/ or upon request from the authors.

### 3.2. Data processing

Diffraction data were processed in *XDS*/*XSCALE* (Kabsch, 2010*b* ▶). *XDS* is currently the only integration software that uses three-dimensional profile fitting, supports PILATUS images and is freely available to academic users. Two sets of parameters were used for integration. The parameters that are not specific to experiment or detector and have differing nondefault values in the sets are listed in Table 2 ▶. Parameter set *A* was generally used throughout this study; parameter set *B* was only used for a comparative analysis where explicitly mentioned to demonstrate the influence of integration parameters on the scaling statistics of fine-sliced data. In both sets the parameter DELPHI, which defines the angular width over which integration parameters are refined in the INTEGRATE step of *XDS*, was chosen such that it was equal to or half the angular width of the wedges of the interleaved data-acquisition scheme. After a first round of integration and refinement of diffraction parameters in the CORRECT step of *XDS*, the refined geometry parameters were applied in a second round  of integration by replacing the file XPARM.XDS with GXPARM.XDS. The intensity estimates obtained in the second round of integration were used in the subsequent processing steps.

Scaling statistics are reported as calculated by *XSCALE*. The precision-indicating merging *R* factor *R*_p.i.m._ was calculated with *SCALA* for data sets not exceeding the maximum number of batches of 5003 using scaled unmerged reflection intensities from *XSCALE* (Weiss, 2001 ▶; Evans, 2006 ▶).

For a subset of data sets the mosaicity as determined by *MOSFLM* is reported (Leslie, 2006*b* ▶). This mosaicity value was obtained by processing the data with default parameters in *iMOSFLM* v.1.0.6b (Battye *et al.*, 2011 ▶). The ‘average mosaicity’ as stated in the *SCALA* log file after using the ‘QuickScale’ feature of *iMOSFLM* is reported.

### 3.3. Anomalous difference Fourier peak heights

Data collected from the thermolysin crystals at the wavelength of the zinc absorption edge were used to calculate anomalous difference Fourier peak heights. A thermolysin model derived from two deposited structures (PDB entries 2g4z and 2tlx; Mueller-Dieckmann *et al.*, 2007 ▶; English *et al.*, 1999 ▶) was refined against each data set using *phenix.refine* (Adams *et al.*, 2010 ▶). Map coefficients for the anomalous difference map as output by *phenix.refine* were used in the *CCP*4 program *FFT* to calculate the maps, peaks were identified and their heights were calculated using *PEAKMAX* (Winn *et al.*, 2011 ▶).

### 3.4. Summation of diffraction images

Diffraction images were summed using the software *TVX*, which is part of the detector-control software of the PILATUS detector supplied by the vendor. The command ‘move a = b + c’ was used to add the pixel values of image *b* and *c* and write the resulting image *a* to disk.

## 4. Results and discussion

### 4.1. Refined mosaicity and Δϕ

**Figure 3:**
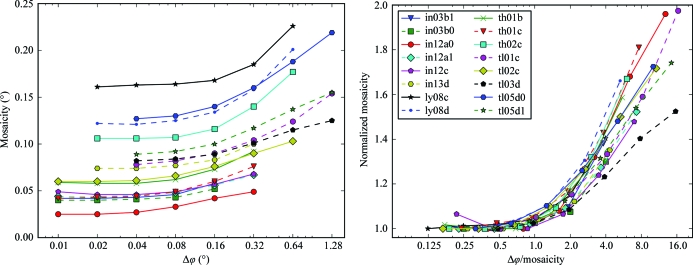
Mosaicity and Δϕ. The mosaicity refined by *XDS* depends on Δϕ. Each line represents a series of data sets collected from the same crystal at increasing Δϕ. In the left panel, the mosaicity as refined by *XDS* and reported in the file CORRECT.LP is plotted on the vertical axis with Δϕ on the horizontal axis. In the right panel, the mosaicity of each data set is normalized to the minimal mosaicity of the corresponding series and plotted against Δϕ divided by the minimal mosaicity.

Analysis of our experimental data shows that the mosaicity refined by *XDS* depends on Δϕ (Fig. 3 ▶). For a series of data sets collected from the same crystal, the mosaicity as reported by *XDS* in the file CORRECT.LP decreases at smaller rotation ranges per image. The mosaicity asymptotically reaches a minimum at Δϕ < σ_ϕ_ for most of the series of data sets. A few series show a small increase in the refined mosaicity for the finest-sliced data set.

The mosaicity is calculated in *XDS* from the angular position of a Bragg maximum and the distribution of the reflection intensity around its maximum (Kabsch, 2010*a* ▶). At finer Δϕ spot centroids are determined more accurately and the reflection intensities are better sampled, which should lead to better estimates of the mosaicity. The lower mosaicity values obtained with finer rotation width are therefore more likely to better reflect the diffraction properties of the crystal in the given experimental setup.

For this and all following analyses of our data we regard the minimal mosaicity value refined by *XDS* for a series of data sets from the same crystal as the crystal’s mosaicity. This seems to be a better choice than the mosaicity value refined from the finest-sliced data set because these data sets might not in all cases be processed with the best accuracy: as discussed later in §[Sec sec4.4.2]4.4.2 the processing results obtained for the finest-sliced data sets can to some extent depend on the processing parameters used.

The minimal mosaicities refined by *XDS* for the crystals used in this study are in the range 0.03° < σ_ϕ_ < 0.16°. If only the widest-sliced data set of each series was available to estimate the mosaicities, these would appear to be in the range 0.06° < σ_ϕ_ < 0.23°, *i.e.* overestimated by a factor of between 1.3 and 2.0.

To put the σ_ϕ_ values obtained with *XDS* into a wider con­text, mosaicities were also calculated with *MOSFLM* for a representative subset of crystals from the widest-sliced data sets (Table 3 ▶). The mosaic spreads determined with *MOSFLM* cover a range of 0.14–0.68° and are two to three times larger than the σ_ϕ_ calculated by *XDS* for the same data sets.

### 4.2. Scaling statistics and Δϕ

**Figure 4:**
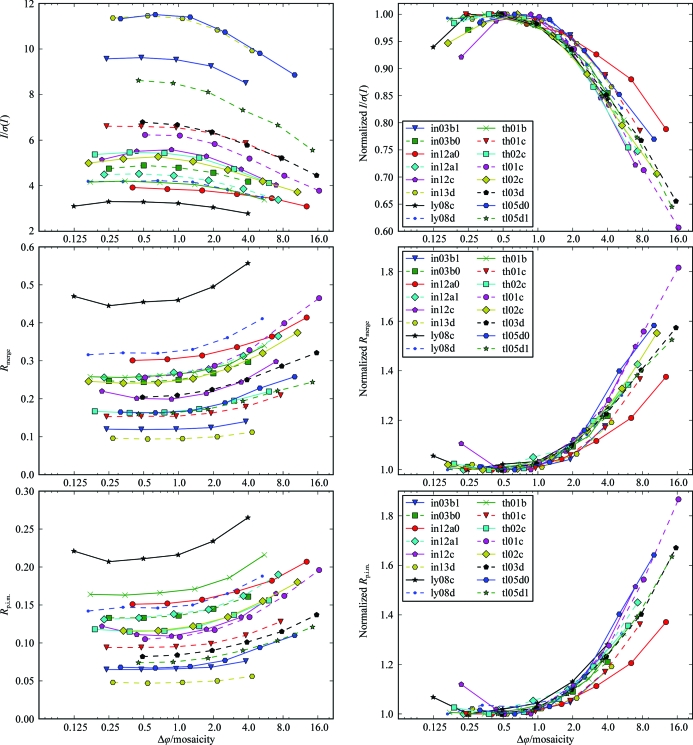
Highest-shell statistics and Δϕ. Statistics of the highest resolution shells of 94 data sets. Each line represents a series of five to seven data sets collected from the same crystal at increasing Δϕ. The statistics on the vertical axis are plotted against Δϕ divided by the minimal mosaicity of the corresponding series on the horizontal axis. In the right panels the statistical value for each data set is normalized against the best value from the same series. The statistics of each series improve for smaller Δϕ up to Δϕ ≃ 0.5σ_ϕ_. For the different series, the scaling statistics are degraded to a varying extent for rotation widths finer than half the mosaicity.

Scaling statistics for the highest resolution shells show a substantial improvement when data are collected at a rotation width smaller than the mosaicity compared with rotation widths several times larger than the mosaicity (Fig. 4 ▶). *I*/σ(*I*) is between 20 and 40% lower for the widest-sliced data set compared with the best fine-sliced data set in the series. For *R*_merge_ and *R*_p.i.m._ the difference between the widest-sliced data set and the best fine-sliced data set of a series can be in the range 20–80%. Several series of data sets exhibit optimal scaling statistics for rotation widths of approximately half the mosaicity, which degrade to a certain extent for finer rotation widths.

**Figure 5:**
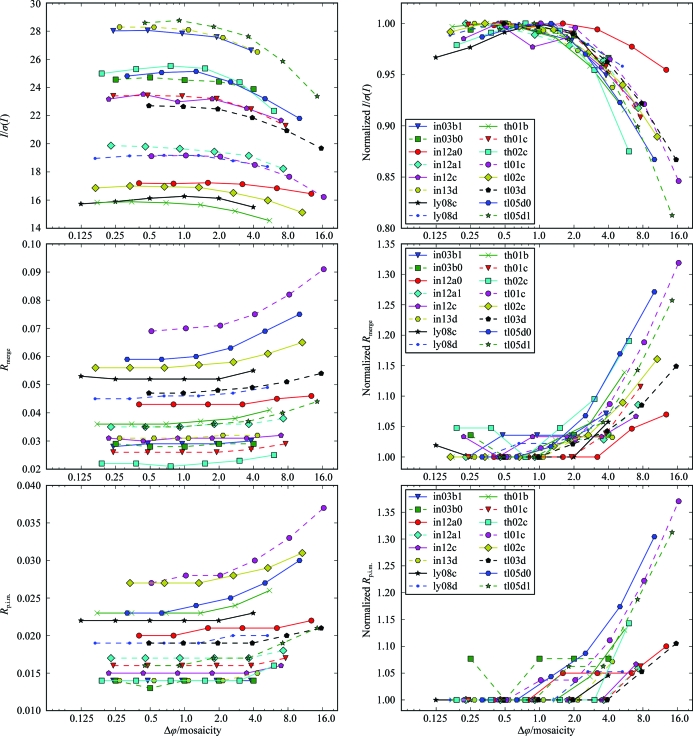
Overall statistics and Δϕ. The figure is similar to Fig. 4 ▶ but shows the overall statistics of each data set. The improvement with smaller Δϕ is less pronounced for the overall statistics than for the highest-shell statistics.

Overall statistics behave similarly to those for the highest shells and show better statistics for finer widths (Fig. 5 ▶). Differences between varying rotation widths are less pro­nounced compared with the highest-shell statistics. This agrees with the theoretical considerations outlined above. From these, we expect that the accuracy of weak high-resolution reflections benefits more from better background separation along ϕ than reflections with stronger intensity. The precision with which the statistics are reported by the scaling software leads to identical values for a number of fine-sliced data sets from the same series for *R*_merge_ and this is more pronounced for lower *R*_p.i.m._ values.

### 4.3. Anomalous signal and Δϕ

Three criteria are used to estimate the anomalous signal in the diffraction data collected from thermolysin crystals at the absorption edge of zinc. The anomalous signal-to-noise ratio 〈Δ*F* ^±^〉/σ(*F*) describes the mean anomalous difference in units of its estimated standard deviation, while the anomalous correlation is the mean correlation factor between two random subsets of anomalous intensity differences (Dauter, 2006 ▶). Both criteria are reported as calculated by *XSCALE* for the overall resolution range of the data. In addition, we calculated the height of the zinc peak in anomalous difference Fourier maps using weighted map coefficients from refinement of a thermolysin model.

**Figure 6:**
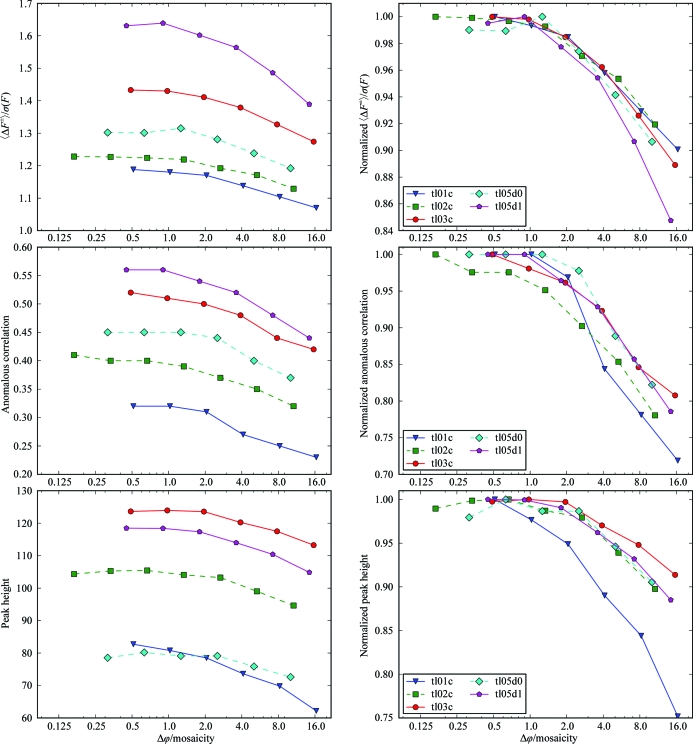
Anomalous statistics and Δϕ. Anomalous signal to noise (top panels), anomalous correlation (middle panels) and anomalous difference Fourier peak heights (bottom panels) for five series of data sets collected at the zinc absorption edge. In the right panels, the statistical value for each data set is normalized against the best value from the same series. All three criteria for the anomalous signal decrease with increasing rotation width.

Analysis of our experimental data over the full resolution range of the data sets shows that a better anomalous signal is obtained from data collected at finer rotation widths (Fig. 6 ▶). The relative differences between the data sets are in the range of roughly 10 to 30%, depending on the series of data sets and criterion evaluated. For some series and criteria a small decrease in anomalous signal can be observed at Δϕ < 0.5σ_ϕ_.

### 4.4. Influence of integration parameters

#### 4.4.1. Sampling of reflection profiles

**Figure 7:**
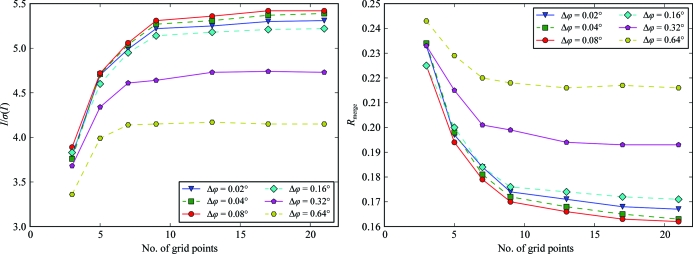
Sampling of reflections for integration. The data sets of series th02c with rotation widths from 0.02 to 0.64° were integrated with a varying number of grid points used to represent reflection profiles in *XDS*. *I*/σ(*I*) and *R*_merge_ of the highest resolution shell from a data set with the specified Δϕ are plotted against the number of grid points used in *XDS*. Scaling statistics for the widest-sliced data set do not improve when more than nine grid points, which is the default value of *XDS*, are used. The statistics for fine-sliced data sets improve when using up to 21 grid points, which is the maximum value possible in *XDS*.

The data sets of series th02c with rotation widths from 0.02 to 0.64° were integrated using a varying number of grid points to represent reflection profiles in *XDS*. Scaling statistics of all data sets deteriorated strongly when fewer than nine grid points, which is the default value in *XDS*, were used (Fig. 7 ▶). The statistics of the widest-sliced data set did not improve when more points were used, but the statistics for fine-sliced data sets improved slightly when using up to 21 grid points, which is the maximum value possible in *XDS*.

Three-dimensional reflection profiles are represented in *XDS* on a grid in a coordinate system specific for each reflection. In the procedure of representing and fitting profiles, the intensity observed on an image covering a certain rotation range is mapped onto the grid points representing the corresponding rotation range in the reflection-specific grid (Kabsch, 2010*a* ▶). A low number of grid points leads to a coarse representation of the reflection profiles *in silico* and to an inaccurate estimation of the reflection intensities. The default of nine grid points is sufficient for wide-sliced diffraction data for an appropriate representation of the observed intensity distribution of the diffraction images and an increased number of grid points does not lead to better scaling statistics. For small oscillation widths the reflection profiles are densely sampled in the diffraction data along ϕ. In this case, the default of nine grid points does not seem to be fully sufficient to effectively represent the dense experimental sampling of the profile *in silico* and better scaling statistics can be observed when the number of grid points is increased.

#### 4.4.2. Highly fine-sliced diffraction data and different sets of integration parameters

**Figure 8:**
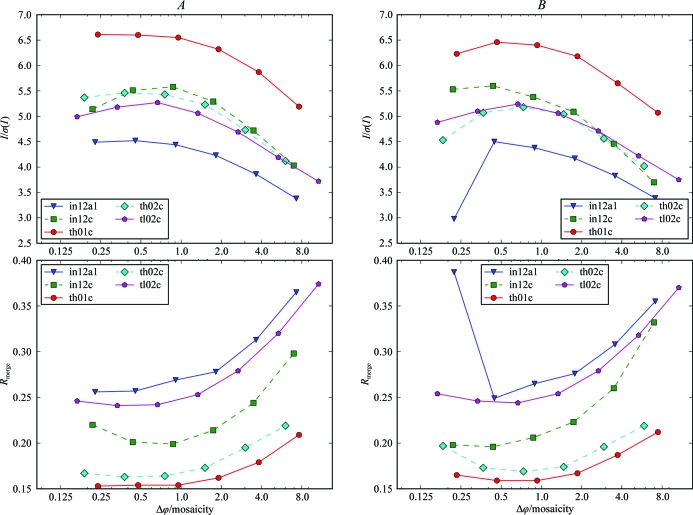
Comparison of two sets of integration parameters. Two different sets of integration parameters (left panels, parameters *A*; right panels, parameters *B*) were used to process the data from five series of data sets. Statistics for *I*/σ(*I*) (top panels) and *R*_merge_ (bottom panels) of the highest resolution shell for each data set are shown. For highly fine-sliced data, better scaling statistics can be obtained with parameters *A* for most data sets.

Several series of data sets exhibit optimal scaling statistics for rotation widths of approximately half the mosaicity, which degrade to a certain extent for finer rotation widths (see §[Sec sec4.2]4.2 and Fig. 4 ▶). Possible reasons for the poorer scaling statistics of the data sets with Δϕ < 0.5σ_ϕ_ are a longer relative dead time per image or experimental errors originating from effects such as beam intensity and position fluctuations or cryocooling-induced sample vibration, which might be averaged on wider sliced images with longer exposure. Moreover, the photon counts in each individual image decrease with finer slicing because the total intensity is distributed over an increasing number of frames. The final scaling statistics, however, do not depend only on the quality of the diffraction data but also on the processing software, which needs to derive accurate intensity estimates from the data. In the previous section, we have seen how a single parameter of the processing software can influence the scaling statistics depending on the rotation width per image used for data acquisition (§[Sec sec4.4.1]4.4.1 and Fig. 7 ▶). In addition, results were obtained which demonstrate that a complex interplay of processing parameters can arise when highly fine-sliced data sets are integrated. When using a different set of processing parameters, set *B* as described in Table 2 ▶, some of the highly fine-sliced data sets show markedly different scaling statistics (Fig. 8 ▶). We processed a representative subset of five series of data sets with both sets of processing parameters. Four of the five series exhibit poorer scaling statistics for the highest resolution shell of the finest-sliced data set with Δϕ < 0.25σ_ϕ_ when processed with parameter set *B*. For these four data sets, scaling statistics for Δϕ > 0.5σ_ϕ_ are virtually unaltered. Series in12c (Fig. 8 ▶, Table 4 ▶) behaves in an opposite way to the other four series. It exhibits better scaling statistics with parameters *B* for Δϕ < 0.5σ_ϕ_ and poorer statistics for Δϕ > 0.5σ_ϕ_. For series in12a1 the scaling statistics of the finest-sliced data set degrade dramatically, indicating a serious problem when integrating this data set with parameters *B*.

Of the parameters in which parameter sets *A* and *B* differ, REFINE­(IDXREF) should not have any effect on the final scaling statistics. This parameter determines how the geometry of the diffraction experiment in the indexing step of *XDS* is refined. The initial geometry is then used at the start of the first integration round. In this study, however, the final intensity estimates are derived from a second round of integration that starts with the diffraction geometry as determined after the first round of integration. The influence of the number of grid points that is used to represent reflection profiles has been demonstrated above. However, when exchanging only the values of the grid-point parameters between sets *A* and *B* the results still differ markedly between the two sets of parameters. This is also the case when only the values of REFINE(INTEGRATE) and MINIMUM_ZETA are altered (results not shown). Therefore, the different results obtained with two sets of parameters cannot be attributed to a single parameter but originate from a combination of several parameters. It is not easily possible to fully explore and understand the complex parameter space, but it should be noted that the results obtained when processing highly fine-sliced data sets can strongly depend on the processing parameters used. One should therefore also consider the processing software and the parameters used for integration of the diffraction data when observing poorer statistics for highly fine-sliced data in addition to potentially poorer diffraction data.

### 4.5. Summation of fine-sliced data

The inaccuracy of the intensity estimates obtained from the integration software is one cause of the observation of poorer scaling statistics with highly fine-sliced data, as demonstrated above. Nonetheless, the quality and the accuracy of the actual diffraction data might also be a reason for the observation of poorer statistics. For perfect fine-sliced data it can be expected that adding images over a certain rotation range will give identical results to collecting the data in this rotation range as a single wide-sliced image. Nonperfect data, *i.e.* those impaired by any error associated with acquiring a large number of fine-sliced images, will give poorer results when added up com­pared with collecting the same rotation range in a single wide image.

We used a summation procedure to evaluate the quality of the highly fine-sliced diffraction data compared with wider sliced data. The pixel values of two consecutive images in the finest-sliced data set were summed to obtain an image corresponding to twice the rotation width of the input images. The summed data set obtained in this way was then again summed to acquire a data set with four times the rotation width of the experimental images from the first step. This was repeated to obtain all summed data sets with rotation widths per image of the data sets that were experimentally acquired. We applied this procedure to five representative series of data sets.

**Figure 9:**
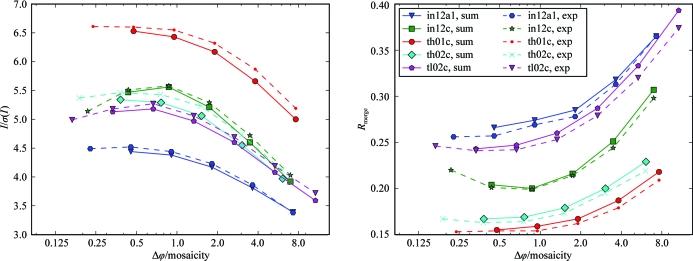
Summation of fine-sliced data. Diffraction images from the finest-sliced data set of five different series were summed to generate images which correspond to experimentally collected images at larger Δϕ. Highest shell statistics of the experimental image data (dashed lines) and summed images data (solid lines) are shown.

The scaling statistics of the highest resolution shells of the summed and experimentally acquired data sets are shown in Fig. 9 ▶. Data obtained by summation of the finest-sliced images give generally poorer scaling statistics than the experimental data of the same rotation width. For series in12a1 and th02c all summed data sets gave poorer statistics than the finest-sliced data, while for the experimental data the second finest data set, collected with a Δϕ of approximately 0.5σ_ϕ_, gave the best scaling statistics. This demonstrates that the diffraction data collected with Δϕ ≃ 0.5σ_ϕ_ are of optimal quality, which degrades upon acquiring finer sliced images. The poorer quality of the finest-sliced data might be caused by a longer relative dead time per image.

Series in12c and tl02c showed best scaling statistics for data in the range 0.5–1Δϕ/σ_ϕ_ for both the summed and the experimental images. It should be noted here that for these two series summation of the finest-sliced images (twofold, fourfold and eightfold summation for in12c, twofold and fourfold summation for tl02c) leads to better scaling statistics than processing the finest-sliced images without summation. Since the summed images are based on the same experimental data, the better scaling statistics for the summed images can only be attributed to the processing software, which seems to derive more accurate intensity estimates from the summed images. A possible explanation for this observation is that summing the finest-sliced images results in poorer diffraction data, but the reflection intensities can be estimated more accurately by the integration software from summed images with larger rotation width. In summary, the conclusion from these results is that the poorer scaling statistics observed for Δϕ < 0.5σ_ϕ_ originate both from poorer quality of the highly fine-sliced data acquired at short exposure times and the intensity estimation by the integration software.

## 5. Conclusions

The theoretical and practical advantages of fine-slicing have been demonstrated previously by Pflugrath (1999 ▶), who compared a wide-sliced and a fine-sliced data set collected from a single crystal using a CCD detector. Based on the experimental results and comprehensive theoretical considerations, a fine-slicing strategy was proposed. Low-mosaicity crystals should be collected with a Δϕ of 0.5°. When the observed reflecting range, which is the definition of mosaicity used in this paper, is larger than 1° a Δϕ of half the reflecting range is recommended. These recommendations are tailored towards CCD detectors and take readout noise and shutter jitter into account, which prevent better quality of finer sliced data.

We demonstrate in this investigation that fine-slicing can be fully exploited with noise-free pixel detectors that record diffraction data in continuous rotation. The quality of integrated diffraction data reliably improves with finer sliced data down to Δϕ ≃ 0.5σ_ϕ_. At this relative rotation width the best scaling statistics are obtained and robust and accurate integration of the fine-sliced data is assured. When the rotation width is less than 0.5σ_ϕ_ poorer statistics may be obtained. Our results indicate that the optimum at 0.5σ_ϕ_ can be attributed to both accurate intensity estimation by the integration software and the poorer quality of highly fine-sliced data collected at very small rotation widths and short exposure times. The influence of the exposure time and relative dead time on data quality need to be further elucidated in future systematic investigations. The influence of the integration software and algorithms should also be further investigated. However, *XDS* is currently the only integration software that uses three-dimensional profile fitting, supports parallelized processing of PILATUS images and is freely available to academic users. Fine ϕ-slicing should also improve the accuracy of intensity estimates of strong reflections because of a more constant count rate over the rotation range and improved count-rate correction (Kraft *et al.*, 2009 ▶). In our data, we did not observe a conclusive correlation between the rotation width and the accuracy of intensity estimates of strong reflections in the low-resolution shells. Strategies for obtaining optimal low-resolution data, such as balancing of dose rate against redundancy, will be the subject of separate future studies. Some initial experiments on this subject have been performed and the preliminary results indicate that lowering the flux in a series of data sets generally results in better scaling statistics for the low-resolution shells.

Our test cases cover crystals with a mosaicity of σ_ϕ_ between 0.03 and 0.16° and a high-resolution limit of the data between 1.20 and 1.95 Å. The crystals used in this study cover more than a fivefold range of mosaicities. Within this range, we observe a uniform distribution of the normalized scaling statistics with respect to Δϕ/σ_ϕ_. It should also be noted that the mosaicities stated here appear to be low because they would be estimated to be higher by a factor of between 1.3 and 2.0 if they were calculated from wide-sliced data and because σ values are reported. When calculating the mosaic spread with *MOSFLM* from wide-sliced images the mosaicity of the crystals used is as high as 0.68°.

Fine-slicing could be particularly advantageous when the diffraction limit of the crystal lies in the region of the solvent ring of background scatter. A better separation along ϕ from the proportionally stronger background in this region could have a stronger effect than in background regions at higher resolution. A stronger advantage of fine-slicing owing to improved background separation can also be expected for crystals that exhibit poorer diffraction and higher background scatter than the well diffracting test crystals in this study. This should generally be the case for small crystals that are frozen in a larger drop of surrounding solvent, with a high solvent content or that exhibit diffuse scattering or a high Wilson *B* factor. These problems are frequently encountered with crystals of membrane proteins or large macromolecular complexes (Mueller *et al.*, 2007 ▶; Carpenter *et al.*, 2008 ▶). However, some poorly diffracting crystals may exhibit irregular spot profiles because of complex mosaic structures. In such cases the observations made with the crystals used in this study might not be valid.

Ideally, our investigations will be complemented in the future by similar studies with crystals of poorer diffraction quality. However, systematic studies with poorly diffracting crystals are intrinsically very difficult. Alternatively, the large amount of systematically collected data acquired for this study can be used to validate the results obtained using simulated diffraction data generated with programs such as *MLFSOM* or *SIM_MX* (Holton, 2008 ▶; Diederichs, 2009 ▶). Diffraction properties and data-collection parameters that are not easily accessible in a systematic experimental study could be investigated based on a validation of the simulation software.

In summary, we recommend any user of a noise-free hybrid pixel detector to collect data at a rotation width per image of half the mosaicity, with the σ_ϕ_ mosaicity definition as used by *XDS*. Obtaining an accurate mosaicity estimate might not be straightforward because of variations between different crystals or an overestimation of the mosaicity from wide-sliced data. However, an accurate mosaicity estimate is not overly important. If the estimate should deviate by a factor of two, data close to the optimum will still be acquired. In contrast, collecting wide-sliced data will degrade the overall, high-resolution and anomalous statistics. This will decrease the maximum resolution of a data set when applying a certain cutoff criterion such as 2〈*I*/σ(*I*)〉 in the highest resolution shell or make attempts at experimental phasing less likely to be successful. Moreover, fine-slicing minimizes the risk of overlaps, which may further reduce the useful resolution of the collected data.

Different applications of the diffraction data such as molecular replacement, anomalous phasing, high-resolution refinement or ligand searching usually require different data-collection strategies (Dauter, 2010 ▶). Fine-slicing, however, generally improves the quality of the acquired data and should be applied in all of these scenarios.

## Figures and Tables

**Table 1:** Crystallization and cryoprotection conditions

	Insulin	Lysozyme	Thaumatin	Thermolysin
Sample solution	20 mg ml^−1^ in 20 m*M* NaH_2_PO_4_/Na_2_HPO_4_ pH 10.3, 2 m*M* EDTA	50 mg ml^−1^ in 50 m*M* sodium acetate pH 4.5	30 mg ml^−1^ in 20 m*M* HEPES pH 7.0	100 mg ml^−1^ in 50 m*M* MES pH 6.0, 45% DMSO, 1.0–1.1 *M* NaCl
Reservoir solution	200–300 m*M* NaH_2_PO_4_/Na_2_HPO_4_ pH 10.2, 2 m*M* EDTA	50 m*M* sodium acetate pH 4.5, 1 *M* NaCl, 20–30% PEG 5000 MME	50 m*M* HEPES pH 7.0, 15% glycerol, 0.9–1.1 *M* sodium/potassium tartrate	1.4–1.5 *M* ammonium sulfate
Cryoprotection	Reservoir solution containing 30% glycerol	Reservoir solution containing 30% PEG 400	Reservoir solution containing 30% glycerol	Fomblin YR-1800 after storage in 25 m*M* MES pH 6.0, 0.5 *M* NaCl, 1 m*M* CaCl_2_

**Table 2:** *XDS* processing parameters

Parameter	Parameter set *A*	Parameter set *B*
REFINE(IDXREF)	ALL	BEAM AXIS ORIENTATION CELL
REFINE(INTEGRATE)	ORIENTATION	BEAM ORIENTATION CELL
NUMBER_OF_PROFILE_GRID_POINTS_ALONG_ALPHA/BETA	15	9
NUMBER_OF_PROFILE_GRID_POINTS_ALONG_GAMMA	15	9
MINIMUM_ZETA	0.15	0.05

**Table 3:** Mosaicities determined with *XDS* or *MOSFLM*

Crystal	in12a1	in12c	ly08c	th01c	th02c	tl02c
*XDS* σ_ϕ_ (°)	0.07	0.07	0.23	0.08	0.18	0.10
*MOSFLM* mosaic spread (°)	0.18	0.14	0.68	0.20	0.48	0.31
Ratio, *MOSFLM*/*XDS*	2.6	2.0	3.0	2.5	2.7	3.1

**Table:** Values in parentheses are for the highest resolution shell.

Data-set series	in03b0	in03b1	in12a0	in12a1	in12c	in13d	ly08c	ly08d
Δϕ† (°)	0.01–0.16 {0.01}	0.01–0.16 {0.01}	0.01–0.32 {0.01}	0.01–0.32 {0.01}	0.01–0.32 {0.02}	0.02–0.32 {0.02}	0.02–0.64 {0.02}	0.02–0.64 {0.04}
Exposure time† (s)	0.10–1.60 {0.10}	0.15–2.40 {0.15}	0.20–6.40 {0.20}	0.20–6.40 {0.20}	0.20–6.40 {0.40}	0.08–1.28 {0.08}	0.10–3.20 {0.10}	0.10–3.20 {0.20}
Readout time (ms)	5.0	5.0	5.0	5.0	5.0	5.0	5.0	5.0
Transmission (%)	0.3	2.6	0.03	0.1	0.1	5.1	0.1	0.1
Wavelength (Å)	1.000	1.000	1.000	1.000	1.000	1.000	1.000	1.000
Space group	*I*23	*I*23	*I*23	*I*23	*I*23	*I*23	*P*4_3_2_1_2	*P*4_3_2_1_2
Unit-cell parameters								
*a* = *b* (Å)	78.19	78.26	78.20	78.17	78.29	78.20	78.48	79.00
*c* (Å)	78.19	78.26	78.20	78.17	78.29	78.20	36.89	36.99
α = β (°)	90	90	90	90	90	90	90	90
γ (°)	90	90	90	90	90	90	90	90
Mosaicity‡ (°)	0.04	0.04	0.03	0.04	0.05	0.07	0.16	0.12
Resolution (Å)	30.0–1.20 (1.27–1.20)	30.0–1.20 (1.27–1.20)	30.0–1.30 (1.38–1.30)	30.0–1.20 (1.27–1.20)	30.0–1.20 (1.27–1.20)	30.0–1.20 (1.27–1.20)	30.0–1.20 (1.27–1.20)	30.0–1.20 (1.27–1.20)
No. of reflections	126642 (16561)	123388 (16665)	102810 (16149)	126094 (16668)	125904 (16574)	132051 (17145)	234628 (30508)	240765 (30835)
No. of unique reflections	24886 (3801)	24925 (3830)	19673 (3187)	24634 (3583)	25038 (3859)	24516 (3491)	36481 (5542)	36860 (5458)
Completeness (%)	99.6 (98.7)	99.5 (99.0)	99.7 (99.5)	98.7 (93.0)	99.7 (99.4)	98.1 (90.3)	99.7 (98.3)	99.2 (95.7)
Multiplicity	5.1 (4.4)	5.0 (4.4)	5.2 (5.1)	5.1 (4.7)	5.0 (4.3)	5.4 (4.9)	6.4 (5.5)	6.5 (5.6)
〈*I*/σ(*I*)〉	24.6 (4.8)	28.0 (9.6)	17.2 (3.9)	19.9 (4.5)	23.5 (5.5)	28.3 (11.4)	15.7 (3.1)	19.1 (4.2)
*R*_merge_ (%)	2.9 (24.7)	2.8 (12.0)	4.3 (30.1)	3.5 (25.6)	3.0 (20.1)	3.1 (9.6)	5.3 (47.0)	4.5 (32.1)
*R*_p.i.m._ (%)	1.4 (13.3)	1.4 (6.5)	2.0 (15.1)	1.7 (13.1)	1.5 (11.1)	1.4 (4.8)	2.2 (22.1)	1.9 (14.7)

**Table:** 

Data-set series	th01b	th01c	th02c	tl01c	tl02c	tl03d	tl05d0	tl05d1
Δϕ† (°)	0.01–0.32 {0.02}	0.01–0.32 {0.01}	0.02–0.64 {0.02}	0.04–1.28 {0.04}	0.01–0.64 {0.01}	0.04–1.28 {0.04}	0.04–1.28 {0.04}	0.04–1.28 {0.04}
Exposure time† (s)	0.10–3.20 {0.20}	0.10–3.20 {0.10}	0.08–2.56 {0.08}	0.10–3.20 {0.10}	0.08–5.12 {0.08}	0.10–3.20 {0.10}	0.10–3.20 {0.10}	0.10–3.20 {0.10}
Readout time (ms)	5.0	5.0	5.0	3.5	3.5	3.5	3.5	3.5
Transmission (%)	2.6	1.0	2.6	0.2	0.33	1.03	1.03	0.71
Wavelength (Å)	1.000	1.000	1.000	1.282	1.282	1.282	1.282	1.282
Space group	*P*422	*P*422	*P*422	*P*6_1_22	*P*6_1_22	*P*6_1_22	*P*6_1_22	*P*6_1_22
Unit-cell parameters								
*a* = *b* (Å)	57.83	57.83	57.88	92.95	92.63	93.09	92.86	92.84
*c* (Å)	150.28	150.16	150.08	130.04	129.44	130.59	130.31	129.87
α = β (°)	90	90	90	90	90	90	90	90
γ (°)	90	90	90	120	120	120	120	120
Mosaicity‡ (°)	0.06	0.04	0.11	0.08	0.06	0.08	0.13	0.09
Resolution (Å)	30.0–1.25 (1.33–1.25)	30.0–1.20 (1.27–1.20)	30.0–1.20 (1.27–1.20)	30.0–1.80 (1.91–1.80)	30.0–1.60 (1.70–1.60)	30.0–1.60 (1.70–1.60)	30.0–1.95 (2.07–1.95)	30.0–1.60 (1.70–1.60)
No. of reflections	237722 (40738)	261787 (33574)	260506 (33102)	423171 (68894)	437929 (72134)	592939 (98771)	290615 (50729)	441713 (72149)
No. of unique reflections	70481 (11888)	74403 (9849)	79622 (12093)	58266 (9501)	81727 (13476)	82667 (13593)	39617 (7125)	78316 (12598)
Completeness (%)	98.4 (99.6)	92.3 (79.6)	98.6 (97.7)	100.0 (100.0)	99.7 (98.9)	98.9 (97.7)	86.4 (94.9)	94.7 (91.7)
Multiplicity	3.4 (3.4)	3.5 (3.4)	3.3 (2.7)	7.3 (7.3)	5.4 (5.4)	7.2 (7.3)	7.3 (7.1)	5.6 (5.7)
〈*I*/σ(*I*)〉	15.9 (4.2)	23.4 (6.6)	25.0 (5.4)	19.1 (6.2)	16.9 (5.0)	22.7 (6.8)	24.8 (11.3)	28.6 (8.6)
*R*_merge_ (%)	3.6 (25.6)	2.6 (15.3)	2.2 (16.7)	6.9 (25.6)	5.6 (24.6)	4.7 (20.4)	5.9 (16.5)	3.5 (16.0)
*R*_p.i.m._ (%)	2.3 (16.3)	1.6 (9.4)	1.4 (11.8)	2.7 (10.5)	n/a	1.9 (8.2)	2.3 (6.8)	1.6 (7.4)

† The range of rotation angles and exposure times per image used in the series of data sets from this crystal. The values in braces are the rotation angle and exposure time of the reference data set, for which unit-cell parameters and scaling statistics are reported in the table.

‡ Mosaicity determined by *XDS* as standard deviation of the reflection range. The value of the data set with the lowest mosaicity is reported.
